# Effect of size-graded and polydopamine-coated halloysite nanotubes on fundamental properties of low-density polyethylene nanocomposite film

**DOI:** 10.55730/1300-0527.3547

**Published:** 2023-02-03

**Authors:** Cüneyt Erdinç TAŞ

**Affiliations:** 1 Materials Research Institute, Technological University of the Shannon Midlands Midwest, Athlone, Ireland; 2 Department of Material Science and Nanoengineering, Faculty of Engineering and Natural Science, Sabancı University, İstanbul, Turkey

**Keywords:** Low-density polyethylene, halloysite nanotubes, polydopamine, mechanical properties, degree of crystallinity

## Abstract

In this study, some of the critical fundamental properties, which are holding importance in usage areas, of low-density polyethylene (LDPE) film were studied by embedding size-graded and polydopamine-coated halloysite nanotubes into the polymer matrix. This concept evaluated the importance of the well-dispersion of nanoparticles in the composite system and interfacial adhesion between nanofiller and polymer matrix on the degree of crystallinity and mechanical properties. For this purpose, halloysite nanotubes, coated with polydopamine and size graded afterward, were integrated into the LDPE matrix by the twin-screw extrusion process, following which, nanocomposite films were prepared by film-blown technique. Both effects of halloysite nanoparticles, having the polydopamine layer on their surface and size-graded, on properties such as mechanical strength, thermal feature, and degree of crystallinity, of those directly acting on the usage goals of LDPE-based films, were tested.

## 1. Introduction

LDPE is an important thermoplastic polymer that contains long-chain branches throughout the polymer backbone. Since it presents many substantial properties such as high clarity, flexibility, chemical, and radiation resistance, crack and fatigue strength, low moisture permeability, also being tasteless and odorless; it is used in a wide range of application areas such as packaging material, bottle, cap, and underpads for hospital beds [1]. These large application areas make the LDPE considerable material in terms of improving their critical properties like mechanical endurance.

One of the most important approaches to reinforce the mechanical properties of a composite system is the dispersion of nanoparticles into the polymer matrix [2]. For this purpose, different nanoparticles have been tried to improve the mechanical properties of a composite such as carbon nanotube (CNTs) [3], graphene [4], ZnO [5], SiO_2_ [6], and Ag [7]. Among these nanoparticles, halloysite nanotubes (HNTs) have a notable place for use as reinforcer filler on polymeric nanocomposite films [8] due to their intrinsic nanostructure, high thermal stability, acceptable mechanical strength, relatively low cost, and biocompatibility properties [9]. In the literature, there are different examples of using HNTs as mechanical property reinforcers for various nanocomposite films as their natural [10–14], or modified state [15–18]. However, there have been limited studies directly focusing on LDPE nanocomposite films [19,20].

To investigate and improve the mechanical properties of nanocomposite film, two main parameters should be considered: First, as known that well dispersion of nanoparticles into a polymer system is the key factor in improving the properties of composite system [21]. Therefore smaller-sized and agglomeration-free nanoparticles should be provided to disperse them into a polymer matrix successfully [22]. Secondly, because poor adhesion lowers the thermal and mechanical properties of composites [23,24], creating a good adhesion between the clay-polymer interface at the nanolevel becomes another remarkable issue to affect the mechanical properties of film composite, positively. Besides, the dispersion of HNTs into the polymer matrix properly ensures the improvement of other important characteristics of packaging material such as enhancing barrier properties which is a critical issue for being used of the material in food packaging applications [25]. Thus, the issue of well-dispersion of nanoparticles into the packaging film should be focused on for being a multifunctional packaging product including its degree of crystallinity which is directly related to barrier property.

In this study, nanocomposite films were designed considering these two main approaches explained above. HNT nanotubes were coated with polydopamine (PD), which has strong adhesive properties, the ability of coating the whole surface of the material, and perfect biocompatibility [26]. Prepared PD-coated HNTs (PDHNTs) were separated into three different size grades as detailedly explained in our previous study [22]. Polydopamine-coated HNTs with different sizes and agglomeration grades were embedded into the LDPE matrix for the concentration of 1% and nanocomposite films were prepared with film blown technique. The effect of polydopamine coating and size dispersion on the mechanical and thermal properties of LDPE nanocomposite films were investigated by thermal and mechanical instrumentation methods.

## 2. Materials and methods

### 2.1 Chemicals

Low-density polyethylene (LDPE) powders (PETİLEN F2-12) and raw HNTs were supplied by PETKİM Petrokimya A.Ş. and Sigma-Aldrich Inc., respectively. Ultrapure Tris base (Tris(hydroxymethyl)aminomethane) and 3-hydroxytyramine hydrochloride (Dopamine) were purchased from MP Biomedicals, LLC, and Acros Organics Inc., respectively. In all necessary steps of nanoparticle preparation, Milli-Q purified water was utilized.

### 2.2 Coating of HNT surface with polydopamine

PDHNTs were prepared by using the traditional PD coating method [27–29]. As the protocol steps, first, a certain amount of raw HNTs was dispersed in distilled water via ultrasound sonication to create a well-dispersion of the HNTs in the aqueous medium. For this purpose, the concentration of the HNT/aqueous medium was adjusted to 10mg/mL, and ultrasound sonication at 100% amplitude (120W-5 s pulse on and 2 s pulse off) was applied to the system in an ice bath for 30 min. Following the dispersion step, the dopamine monomer (2 mg/mL) was put into the dispersion, and the pH of the system was set to 8.5 with Tris base powder. The dispersion was stirred at 30 °C for 30 min, and PDHNTs were collected from the system at the end of the 30 min with centrifugation at 5000 rpm for 5 min. The prepared PDHNTs were washed with water several times and dried at 50 °C for 24 h in a vacuum oven.

### 2.3 Separation of PDHNTs into three different size grades

PDHNTs were separated into three different grades by preparing a main aqueous system in a concentration of 20 mg/mL. The prepared aqueous system was dispersed by Ultra-TURRAX T18 for 30 min at approximately 11,000 rpm to provide a predispersion, first. Following, the predispersed mixture was transferred to an ultrasound sonicator and ultrasonicated for 45 min in an ice bath with the parameters of 120W/5 s pulse on and 2 s pulse off. Finally, the obtained well-dispersion of PDHNT was centrifuged at 2000–6000–11,000 rpm for 10 min each. At the end of each centrifugation, the precipitated PDHNTs for different grades were collected, and the supernatant part was centrifuged with the next rpm level. Each PDHNT quality, which was precipitated after the centrifugations at 2000, 6000, and 11,000 rpm, was labeled as “grade 1”, “grade 2” and “grade 3”, respectively, and dried in an oven at 100 °C overnight. This section was detailedly examined and explained in the previous work of Tas et al. [22].

### 2.4 Preparation of LDPE-PDHNT nanocomposite films

The mixture of polyethylene and PDHNT powders in the ratio of 99:1 (wt./wt. %) was fed into a twin-screw extruder (Zamac Mercator with a screw diameter of 12 mm and L/D of 40). Powder mixtures were processed at the zone temperature range between 160 °C and 180 °C with the screw speed of 300 rpm. Nanocomposite melt flowing from the extrusion die was cooled in a water bath and pelletized. Obtained pellet form of nanocomposite mixture was transferred to a single screw film blowing machine (Scientific Laboratory Ultra Micro Film Blowing Line Type LUMF-150 with L8-30/C, LabTech Engineering), processed at 150–160 °C with a single screw speed of 80 rpm and blown into 55–65 μm thick films.

### 2.5 Characterization methods

Nicolet IS10 Fourier transform infrared (FTIR) spectroscopy with an ATR system was used for the chemical analysis of samples. The hydrodynamic diameter of samples was determined using a dynamic light scattering (DLS) instrument (Zetasizer Nano - ZS, Malvern Instruments Ltd., UK) at 25 °C at a sample concentration of 2 mg/mL for each sample. The samples were visualized with Zeiss LEO Supra 35VP scanning electron microscopy (SEM). As the sample preparation methods for SEM imaging, 0.1 mg/mL aqueous PDHNT mixture was dispersed by ultrasonication in an ice bath for 2 min (at 75 W with 5 s pulse on and 2 s pulse off), dropped on the silicon wafer and dried. For the imaging of LDPE/PDHNT nanocomposite films, samples were coated with Au-Pd, and images were recorded with a secondary electron detector under a high vacuum at 5 kV. The size distribution of HNT specimens was analyzed using SEM images at 50k magnification by ImageJ software. Mechanical properties of nanocomposite films were examined by Zwick Roell universal testing machine (Z100 UTM), with a load cell of 200 N and a crosshead speed of 12.5 mm min^−1^ according to the ASTM D1708–10 testing method. Dog-bone test specimens had a length of 38 mm and width of 15 mm, a narrow section width of 5 mm, and a grip distance of 22 mm. An average of at least four replicates of each sample was reported. TGA analysis was carried out by Shimadzu Corp. DTG-60H (TGA/DTA) instrument in the temperature range of 30–1000 °C with a rate of 10 °C/min under nitrogen. The thermal properties of samples were investigated by DSC (Thermal Analysis MDSC TAQ2000) with the heating and cooling cycle between 60 °C and 200 °C at a heating/cooling rate of 10 °C/min under nitrogen flow. The DSC parameters were calculated using the TA Universal Analysis Software. The thicknesses of the films were determined by a digimatic micrometer (Mitutoyo Quicmike, no. 99MAB041M). Contact angle studies were performed by the Theta Lite Contact Angle Measurement system with a sessile drop method and an automatic video-based contact angle device. The characterization was done at room temperature and results were reported as averages of the measurements at three different points on the nanocomposite film surface.

## 3. Result and discussion

Raw HNTs which were supplied from any resource can be separated into homogeneous, smaller-size grades, and agglomeration-free levels by applying the three-step separation method. The brief methodology of this technique is that raw HNTs are coated with polydopamine to enhance the hydrophilicity of the nanotube surface and provided colloidal stability in the water medium, firstly. Afterward, polydopamine-coated HNTs are cut by ultrasound sonication to a smaller size, and finally, size-graded PDHNTs are collected by centrifugation at different speeds. With this pathway, homogeneous-smaller-sized and agglomeration-free PDHNTs have been obtained [22].

**Figure 1:**
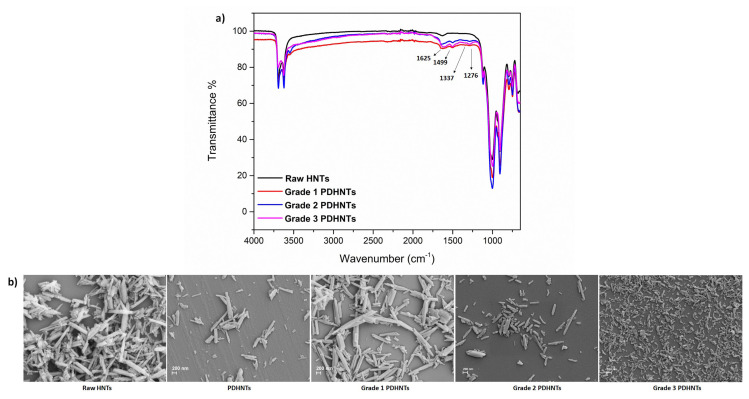
FT-IR spectrum (a) and SEM images (b) of raw HNTs and PDHNTs at different size grade.

Fundamental results of three different-sized PDHNTs were presented in Figure 1 and Table. In Figure 1a, the FT-IR spectrum of raw HNTs and PDHNTs at different size grades were presented. As assigned on the spectrum, indicator peaks of polydopamine coating at 1625 cm^−1^, 1337 cm^−1^, 1499 cm^−1,^ and 1276 cm^−1^ corresponded to -NH bending, symmetric and asymmetric -NH stretching, aromatic C=C bending, and C-N stretching, respectively [30], and the intensity of characteristic peaks of polydopamine coating on HNT surface increased toward grade 3 PDHNTs.

SEM images of raw HNTs and PDHNTs at different size grades were given in Figure 1b. As seen on SEM images, raw HNTs possessed a widescale of size distribution and microsized agglomeration, while distribution and the size length of nanotubes reached the smallest and more homogeneous level from grade 1 PDHNTs toward grade 3 PDHNTs. Also, grade 3 PDHNTs presented a minimum amount of aggregates when compared with other size-graded PDHNTs and raw HNTs.

The amount of polydopamine coating on PDHNTs was calculated by TGA (Table). The amount of PD layer on grade 1, grade 2, and grade 3 PDHNTs was calculated as 1.51 wt %, 1.70 wt %, and 3.63 wt %, respectively. The results apparently exhibited that HNTs having the agglomeration-free form and smaller size were coated with the polydopamine layer in higher percentages than HNTs having larger agglomerated features. This was probably due to having larger surface areas of agglomeration-free HNTs relative to the surface areas of agglomerated HNTs.

In Table, the size distribution of PDHNTs was also presented by scanning electron microscopy (SEM) and dynamic light scattering (DLS) analysis. It was found that raw HNTs showed a wide distribution of nanotube lengths with micronsized aggregates. After the separation protocol, it was clearly seen that the size fractions of PDHNTs formed more homogeneous distributions of sizes with a minimum amount of aggregates. As seen in the hydrodynamic diameters calculated from DLS analysis, the size length of HNTs dropped to 200 nm for grade 3 HNTs. Moreover, statistical analysis of PDHNT length distributions on the SEM images also proved that the wide length distribution of raw HNTs was reached to narrower distributions with shorter average lengths that are grade 2 PDHNTs had shorter nanotubes, and grade 3 PDHNTs presented only individually separated nanotubes of shortest lengths.

Following the fundamental characterizations, obtained PDHNT grades and raw HNTs were integrated into the LDPE matrix. Thus, the effect of size-graded and PD-coated HNTs on the thermal and mechanical properties of LDPE nanocomposite films was examined by focusing on the degree of crystallinity and fundamental mechanical properties such as Young’s modulus, tensile strength, and elongation at break.

**Figure 2:**
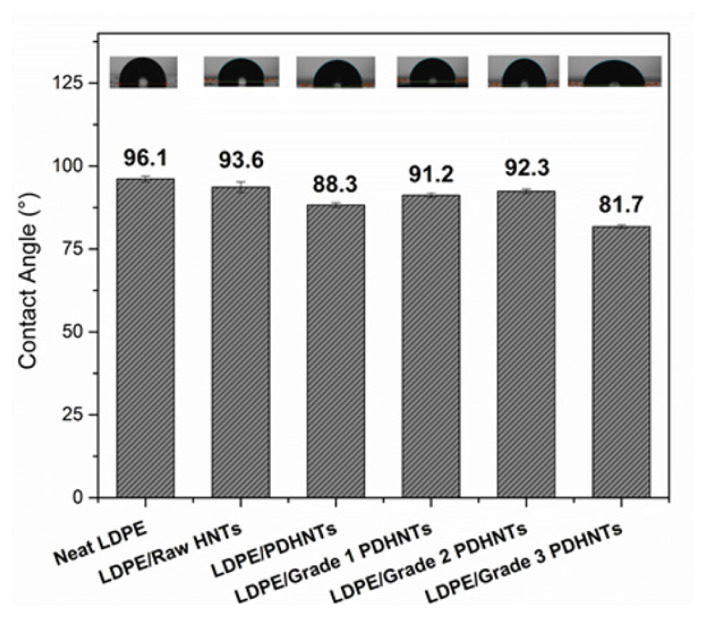
Contact angle comparison between neat LDPE and LDPE nanocomposite films.

Firstly, the influence of PD-coated nanotube incorporation on the surface hydrophilicity of LDPE nanocomposite films was investigated, and comparison results were presented in Figure 2. The integration of raw HNTs into the LDPE matrix decreased the contact angle value due to the hydroxyl character of the HNT surface, thus increasing the surface hydrophilicity of nanocomposite films. The incorporation of PD-coated HNTs decreased the contact angle more than raw HNTs, which was the expected result because of the hydrophilic character of PD coating arising catechol groups on PD structure. The low increment of contact angle values of LDPE/grade 1 PDHNTs and LDPE/grade 2 PDHNTs could be explained by the aggregation of PDHNT nanotubes at different points in the LDPE matrix. Finally, a significant decrease in the contact angle of LDPE/grade 3 PDHNTs nanocomposite films was observed, which could be an important indicator of the well-dispersion of grade 3 PDHNTs into the film matrix and the PD coating amount of HNT surface. Therefore, the hydrophilic character of LDPE/grade 3 PDHNTs nanocomposite film was ascended.

As reported in different studies, the concentration of HNTs in the composite system has a limited amount. When an excess amount of HNTs is added to a composite system, the mechanical properties of the prepared composite start to decrease, probably due to poor dispersion in the polymer matrix. In general, 3% of HNT concentration can be accepted as the maximum HNT filler content for the protection mechanical of parameters [14,31]. Because of this reason, the HNT amount in nanocomposite films was kept at 1% for investigating the size and PD coating effect clearly by avoiding the concentration effect. SEM images of prepared nanocomposite films and their mechanical parameters of them were presented in Figure 2.

**Figure 3:**
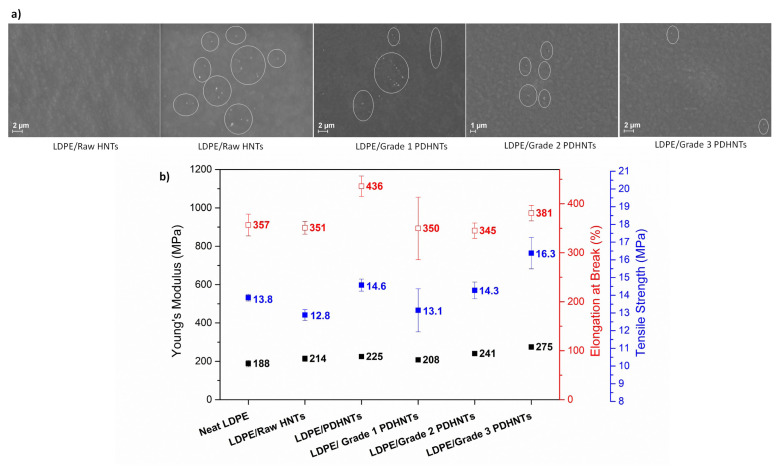
SEM images (a) and mechanical parameters (b) of LDPE/HNTs and LDPE/PDHNTs nanocomposite films.

In Figure 3a, a general view of prepared nanocomposite films at 10k magnification was presented. As marked with white circles on the images, many small chunks were detected in the film matrix. However, these nanotube chunks decreased from grade 1 to grade 3 and obtained a negligible number of chunks in LDPE/Grade 3 PDHNTs nanocomposite film. These results showed that when the size of PDHNT nanotubes was decreased to a smaller level, better integration in the nanocomposite film was provided which was a very important indicator of well-embedding and dispersion of nanotubes into the polymer matrix.

In Figure 3b, Young’s modulus (YM), elongation at break (EB), and tensile strength (TS) values of prepared nanocomposite films were evaluated. An increasing trend was observed for Young’s modulus and TS of nanocomposite films. When compared with neat LDPE, 46% (YM) and 18% (TS) improvement were obtained for LDPE/grade 3 PDHNTs film. For EB values, almost similar and random results were found. The reason for obtaining large error bars that were seen for TS and EB values of LDPE/grade 1 PDHNTs was probably due to poor dispersion and large chunks in the film matrix causing defects on the film matrix, thus resulting in a wide range of results. Moreover, the effect of polydopamine coating on filler performance was compared by using mechanical parameters of LDPE/raw HNTs and LDPE/PDHNTs. It was obviously seen that the TS value of LDPE/PDHNTs had %14 higher than LDPE/raw HNTs, while YM values were almost the same. These improvements in modulus value mostly depend on the dispersion state of nanotubes and interfacial adhesion between filler and matrix due to the strong adhesion character of PD coating [23].

**Figure 4:**
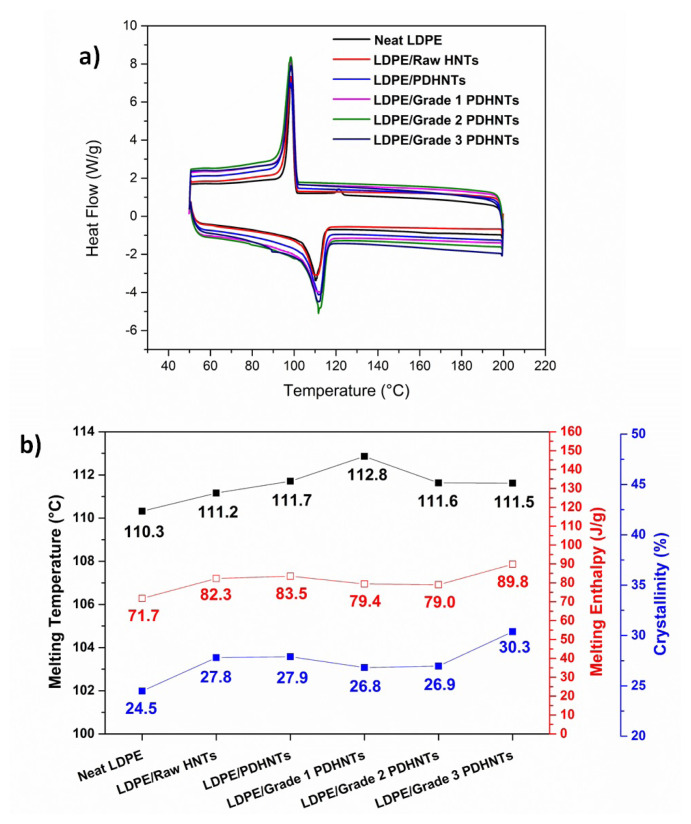
DSC curves (a) and thermal parameters (b) of Neat LDPE, and nanocomposite films.

The contribution of PD-coated HNT fillers on the thermal properties of LDPE was investigated by DSC measurement and results were given in Figure 4. DSC curves of film samples were presented in Figure 4a and melting properties with crystallinity % results were drawn in Figure 3b. The degree of crystallinity % (*X*_c_) was calculated using the equation,


$$X_{\text{c}}=\Delta {H^{o}}_{m}/\Delta H_{m}(1-\text{wt})\times (100),$$

where Δ*H_m_* is the specific melting heat, calculated by the integration of the area under the crystallization peak, Δ*H°_m_* is the theoretical specific melting heat of 100% crystalline PE (293 J g^−1^27 and it is the weight fraction of HNTs in the polymer matrix.

As known that the integration of HNT nanotubes into a polymeric matrix increases the degree of crystallinity of nanocomposite film due to enhancing interaction between nanotubes and polymer matrix, where nanotubes may act as a nucleation site [19]. In this presented study, similar results were obtained as seen in Figure 4b. It was calculated that the degree of crystallinity increased by 13% by being added raw HNTs into the LDPE matrix and showed the same trend for PDHNT, grade 1 PDHNT, and grade 2 PDHNT containing nanocomposite films. For LDPE/grade 3 PDHNT nanocomposite films, the degree of crystallinity was found 24% higher than the neat film that was because of well dispersion and strong interfacial adhesion arising from PD coating. Parallel with the degree of crystallinity results, melting enthalpies exhibited a similar trend, while melting temperatures did not change notably.

In conclusion, the effect of polydopamine coating and size separation of HNTs on the dispersion state of nanotubes into the LDPE-based polymer matrix was analyzed in terms of contribution to the degree of crystallinity and mechanical properties of nanocomposite films, which are important parameters for application areas of LDPE such as food packaging. It was found that improvement of both degrees of crystallinity and the mechanical parameter was higher than results that were obtained in different studies used treated or raw HNTs for various targets[20,24,32–34], especially for LDPE/Grade 3 PDHNTS that used homogeneous-smallest-sized and agglomeration free PDHNTs. Moreover, a comparison of the degree of crystallinity and mechanical properties of the LDPE/raw HNTs with LDPE/PDHNTs (nongraded) displayed positive contributions of PD coating on the improvement of LDPE in terms of examined parameters.

## Figures and Tables

**Table:** Amount of PD coating on HNT surface and analysis of size distribution of raw HNTs and PDHNTs at different size grade.

Sample	PD amount on PDHNTs (wt %)#	Hydrodynamic diameter (d.nm)*	Size length (nm)$
Raw HNTs	-	1417 ± 378	527 ± 25
PDHNTs	5.90	2639 ± 893	416 ± 82
Grade 1 PDHNTs	1.51	514 ± 22	276 ± 17
Grade 2 PDHNTs	1.70	315 ± 9	248 ± 8
Grade 3 PDHNTs	3.63	199 ± 3	132 ± 3

# Calculated with TGA analysis using differences between total weight loss of raw HNTs and PDHNT samples.

* Measured with DLS instrument in water medium.

$ Calculated with SEM images at 50k magnification using ImageJ software.
